# On the Diseases, Etc. of the Joints

**Published:** 1860-09

**Authors:** 


ART. IV.—*On the Diseases and Injuries of the Joints. Clinical and Pathological Observations.* By THOMAS BRYANT, F.R.S., Assistant Surgeon, Clinical Registrar, and Lecturer upon Operative Surgery at Guy's Hospital. 12mo., pp. 273. London, 1859.

THE English language does not possess a great, or even a respectable, work on the diseases and injuries of the joints. The medical profession is indebted, it is true, to a British surgeon for having laid, in a broad and enduring form, our scientific knowledge upon the subject; but the treatise which he published upon it was ushered into the world upwards of forty years ago, and has, therefore, notwithstanding the occasional additions of its illustrious author, become in great degree obsolete. How well the labors of Sir Benjamin Collins Brodie—for we need hardly say it is to him we refer—were appreciated in England and in the United States is known to every one familiar with the history of the literature of the profession within the last third of a century. In both countries it has passed through numerous editions, and has perhaps had as many readers as any monograph that ever appeared in the English language. Nevertheless, the work never was a great work; as a sketch of the diseases of the joints, graphic and true to nature, it was admirable, but beyond this it had no claims to the respect and gratitude of the practitioner. As a full and comprehensive digest of the existing state of the science, it was altogether defective, and for a long time unworthy of the advances, slow as they certainly have been, in this particular branch of the healing art. In comparison with the treatise of Bonnet it is a mere epitome, whose later improvements have failed to realize its earlier promises as an elaborate monograph, destined to occupy a permanent place in our literature. For the last twenty-five years or more, the English surgeon has rested quietly upon his laurels, and permitted a continental brother to snatch from him the chaplet which, in his earlier manhood, had sat so gracefully upon his brow. M. Bonnet, of Lyons, has produced the only great work extant upon the diseases and injuries of the joints. That this work, so creditable to medical literature, and so complete in its design and finish, should still be locked up in the French language, and therefore inaccessible to American and English students, may well be regarded as one of the great marvels of the day. It can only be explained upon the supposition, not that there are not plenty of men in the profession competent to translate it, but that there is no publisher willing to incur the hazard of bringing it out in a suitable form, accompanied as it is by a large atlas of plates, under the idea that the enterprise might not prove to be sufficiently remunerative.

When we say the work of Mr. Bryant is *respectable*, it is all that we can justly claim for it; and yet, in making this remark, we do not wish to be considered as captious or fault-finding. It has decided merit. Indeed, he himself does not claim for it any very exalted position. "It is not to be regarded," says he, "as a complete treatise upon these important and interesting subjects, but simply as the record of much personal investigation, and of some experience." Viewed from this stand-point we feel grateful to Mr. Bryant for his labors in a department where his countrymen have of late accomplished so little, and where American surgeons have not yet even broken ground, as if they were afraid to embark upon the study of a class of affections of which immense numbers of cases are everywhere met with in private and hospital practice, from one year's end to the other. The English writer, who, we suppose, is still a young man, has evidently enjoyed large opportunities for observing articular diseases and injuries, and—what is greatly to his credit—he has made excellent use of them, though not all the use that might have been expected. The principal defect of the work is its want of generalization and the meagreness of detail in his cases, which are seldom anything more than mere rapid outlines of what he saw at the bedside. In the hands of an older practitioner, these cases might have been readily elaborated into a great systematic treatise, more commensurate with the wants of the age and more creditable to English literature. The descriptions of Mr. Bryant of the causes and symptoms of the diseases of which he speaks are in general sufficiently graphic; but his pathological and practical deductions are often strikingly deficient, if not positively in arrear of the existing state of the science. Moreover, his style is not unfrequently awkward, and his language inaccurate or marked by grammatical blunders. These are matters, however, which will doubtless be corrected in future editions, enlarged and improved as they must be by further researches and a better appreciation of the wants of our literature.

The first topic discussed by Mr. Bryant is *synovitis*, which he divides into acute, subacute, chronic, and rheumatic. Not one word is said, so far as we can perceive, of the syphilitic variety of the disease, so liable to occur during the progress of the more severe cases of the tertiary form of that ruthless malady. We regard this as a great oversight, for which it would be difficult to account, inasmuch as articular syphilis must be sufficiently common at Guy's Hospital, of which Mr. Bryant is assistant surgeon. At page 25 he speaks of a form of *synovitis associated with gonorrhœa*, which, it is added, is most obstinate to treat. In the few comments which he makes upon the subject, he alludes to the fact that there are some surgeons who altogether deny the existence of such an affection as an independent lesion, while he states that they are often associated together, and that they are probably produced by the same cause. That the two complaints occasionally coexist, we readily admit, but that the poison of gonorrhœa ever induces rheumatism of the joints, is what we have never been able to believe, simply because we have never, in a practice of many years, had the slightest evidence of it. To come to any other conclusion has always seemed to us exceedingly absurd, seeing that gonorrhœa is universally regarded as a mere local malady. The complication alluded to by the author appears to be much more common in the damp and variable climate of England than in any other country; and we presume the subjects of it are generally persons of intemperate habits and of broken-up constitution, although no mention is made of the fact. Perhaps, too, most of such patients may be affected with a syphilitic taint of the system, either inherited or acquired. Such a supposition, at all events, derives some plausibility from the circumstance that the most efficient treatment, according to the author, is iodide of potassium with iron, remedies which exert little, if any, influence over gonorrhœa, and certainly not much over ordinary cases of rheumatism.

In the *treatment* of synovitis, as laid down by Mr. Bryant, we perceive nothing new or different from what has been inculcated heretofore. Our chief reliance is, of course, upon antiphlogistics, aided by recumbency and absolute rest of the parts, with the repeated application of leeches.

If we have any fault to find here with the author, it is that he does not take blood enough from the general system; but as an excuse for this, he might probably plead the inability of his class of patients to tolerate such spoliation, and, truth to say, it is not very often, even in the more robust subjects, that general bleeding is called for in ordinary cases of the disease. It is only when the attack is uncommonly violent or intractable that the lancet is, as a general thing, at all required. After the malady has been in some degree subdued, mercury comes in play, either in the form of blue pill, calomel, or the gray powder, in combination with Dover's powder, or, what we usually prefer, morphia and antimony, with a view to their diaphoretic and anodyne effects. The bowels should be moved by saline and other cathartics, and the diet should be regulated by the exigencies of each particular case, being abundant and nutritious when the patient is enfeebled, and the reverse when he is strong or plethoric. As the disease declines, recourse may be had to tonics, and to support of the joint by means of strapping. In the subacute form, blisters and iodide of potassium, with iodide of iron, are often serviceable.

In rheumatic and gouty synovitis, our chief reliance is upon remedies which act upon the blood, and thus remove the exciting cause of the disease. Among the best of these remedies are the alkalies, with colchicum and anodynes, saline purgatives, and, in cachectic subjects, tonics. The most reliable topical measures are hot fomentations and blisters in acute attacks, and in chronic the application of dry sulphur beneath a flannel bandage. Lemon-juice is regarded as serviceable by the author; but in our own practice we have never derived any appreciable benefit from its use, however protracted. We have ourselves, after proper depletion in cases of plethora, found the wine of colchicum, in drachm doses, with the addition of from one-half to one grain of morphia, given at bedtime, the most speedily efficacious treatment both in the gouty and in the rheumatic forms of the affection.

When the joint is greatly distended with synovial fluid, the repeated application of blisters, conjoined with minute doses of mercury, will be found to be the most available means. Evacuation of the fluid can only be required when the absorbents are unwilling, or absolutely refuse, to perform their proper functions. When pus forms, the sooner it is let out the better. Its retention will inevitably lead to serious structural changes. We have enjoined the importance of this practice for nearly a quarter of a century, in our annual courses of lectures.

The author treats of what he, in common with Sir Benjamin Brodie and others, calls "the *pulpy and gelatiniform disease* of the synovial membrane." The true character of this lesion has long been a matter of dispute, but there can be little doubt that it is simply a softened, thickened, and degenerated condition of the synovial membrane, in consequence of protracted inflammation, attended with inordinate interstitial deposits of semi-organized plasma. Such alterations may follow upon any long-continued inflammation, whether simple, rheumatic, gouty, strumous, or syphilitic, and are always extremely prone to terminate in complete anchylosis or destruction of the functions of the joint. The most prominent symptoms are great stiffness of the affected articulation, although there is usually considerable motion, slight aching pain, with an occasional dart or stitch, more or less tumefaction of the structures around the joint, with a peculiar doughy, brawny, or inelastic sensation on manipulation, and a marked accumulation of synovial fluid. The general health is ordinarily a good deal impaired. The disease may last for years. Abscesses, followed by intractable sinuses, often form, and greatly aggravate the suffering.

The principal remedies in the pulpy degeneration are mild mercurials, as the gray powder, or the bichloride, and Dover's powder, given with great caution, a properly regulated diet, absolute repose of the joint, as insured by means of pillows, sand-bags, or splints, moderate leeching, and blistering. When the inflammation and swelling have been greatly lessened, the cure is completed by strapping the joint, according to the method originally suggested and so successfully practiced by the late Mr. John Scott, of England, with strips of linen spread with the compound mercurial ointment or the ammoniac and mercurial plaster. Any tendency to anchylosis or contraction is counteracted with the angular screw splint. The general health is improved by tonics and nutritious diet. When the disease is irremediable the affected bone is excised, the worst cases alone being subjected to amputation.

As might be supposed, the diseases of the *articular cartilages* and of the articular extremities of the bones claim a full share of attention in the treatise before us. After briefly adverting to the mode of nourishment of the articular cartilages, the author proceeds to give an account of their diseases, the principal of which are atrophy, granular degeneration, fatty degeneration, and fibrous degeneration. Each of these lesions is described very briefly but sufficiently graphical, and they are regarded as being of a secondary character, or the result merely of an extension of disease from the surrounding parts, especially the bones. The existence of primary disease in the articular cartilages is, it is true, admitted as an occasional occurrence, but that is all. Great exception might, we think, be taken to such a sweeping conclusion, but we pass it by, not having time to do the subject justice. However induced, the malady generally ends in the destruction, partial or complete, of the affected cartilage, and also, as a necessary consequence, in loss of function of the joint. The diagnosis is always obscure, and the treatment antiphlogistic.

The diseases of the *articular extremities of the bones* are generally of an inflammatory character, the morbid action being either acute or chronic, spontaneous or the result of injury, strumous or syphilitic. It may terminate, in the healthy subject, by resolution, and in a similar manner in the cachectic, under proper treatment. The affected bone may become carious, necrosed, or the seat of an abscess. Unless the malady is limited to a particular portion of the bone, the cartilages soon become involved in granular degeneration, and, the effects gradually spreading, the other structures of the joint eventually share a similar fate, followed by complete annihilation of function, if not by loss of life.

Mr. Bryant thinks that the majority of the cases of affections of the articular extremities of the bones, which are described by surgeons as strumous or scrofulous diseases of the joints, depend upon chronic inflammation of the bones. He asserts that it is "quite exceptional to find in any bone that yellow, cheesy material which pathologists so well know as strumous deposit;"—"and if, then, we confine," he adds, "the term strumous disease of a bone, as I believe we should, to such instances only where such a deposit is present, as surgeons we shall seldom have occasion to employ it." We agree entirely with the author in his opinion respecting the inflammatory character of this class of affections; and what he says about the absence of "yellow, cheesy deposit," is unquestionably also true; but such absence does not, according to our notions of the pathology of these diseases, prove that they are not scrofulous; for it is very well known that strumous disorders are liable to occur in various parts of the body, as in the eyes and eyelids, the skin and synovial membranes, without any appreciable deposit, such, for example, as occurs in the lungs in confirmed phthisis. There is a strumous something here, a scrofulous atmosphere, or tubercular dyscrasia, or whatever else we may call it, which leaves its specific impress upon the affected structures, and effectually does its work, and that in a manner altogether different from ordinary inflammation. Coxalgia, white-swelling, psoas abscess, and Pott's disease of the spine, all appertain to this class of lesions, and every intelligent surgeon knows how difficult it is to arrest them when they have once fairly commenced.

The symptoms and diagnosis of these chronic affections are very imperfectly discussed in the pages of Mr. Bryant. The most valuable phenomena, practically considered, are slight but gradually increasing pains of a dull, aching character; impairment of motion, swelling and alteration in the contour of the joint from effusion of synovial fluid and deposits in the extra-articular structures; more or less tenderness on pressure; and startings or twitchings of the limb. The general health, perhaps at first good, ultimately suffers. The disease nearly always occurs in weakly subjects. Mr. Bryant justly cautions practitioners not to disregard the earliest complaint of pain or aching of a joint in delicate strumous children. Parents too often ascribe such suffering to the effects of what are vulgarly called "growing pains," and the consequence is that the disease upon which they depend too frequently escapes attention at a time when alone it admits of thorough eradication.

We find nothing new in respect to the management of this class of affections. Mr. Bryant very properly calls attention to the great necessity of constitutional treatment, regarding it as of paramount importance to supply power to the patient to resist the morbid action and to shake off its effects. For this purpose the different preparations of iron, especially the iodide, in the form of syrup, or the phosphate, in union with phosphate of lime, cod-liver oil, a good diet, and bracing air, are to be employed. Sometimes a mild mercurial course is beneficial, the bichloride and gray powder being the most suitable articles. The most important topical remedies are absolute rest, with elevation of the affected limb, cold or warm applications, according to the relief they confer, and the occasional use of a few leeches when there is much pain, heat, or tenderness. In the more advanced stages of the complaint, strapping and mercurial ointment will be useful. If matter form, it must be promptly evacuated. Necrosed bone must be removed.

The author does not mention, in the treatment either of this or any other form of joint disease, issues made with the actual cautery. From his silence upon the subject, it may be assumed that he has no experience with them; a very singular circumstance, surely, considering the wonderful influence which this mode of counter-irritation exerts over the progress of all chronic articular lesions, particularly over such as are of a strumous nature.

*Anchylosis* and Amputation or Excision form the subjects of the eighth and ninth chapters; but the materials, although in the main sound, are so commonplace that we shall not detain our readers with an account of them. All that Mr. Bryant says upon these topics may be found quite as well expressed in any systematic treatise on surgery, English or continental. The same is true of "Loose Bodies in the Joints," treated of in the next chapter. When surgical interference is rendered necessary, Mr. Bryant is, of course, an advocate of subcutaneous extraction, an operation which he describes in connection with the name of Professor Syme, without any allusion to that of M. Goyrand, of Aix, who either preceded the Scotch surgeon, or hit upon the same process about the same time.

A short chapter is devoted to "*Inflammation of the Cellular Tissue*

*external to the Joints*, and to *Bursitis*," and is one of the most interesting in the book. The twelfth chapter, and the last which is appropriated to articular diseases, properly so called, treats of historical affections, the whole subject of which is dispatched in about three pages.

Part Second of the work occupies nearly one hundred pages, being taken up with the consideration of the injuries of the joints, as sprains, wounds, and dislocations, every articulation receiving some share of attention. A perusal of this portion of the book will prove highly instructive to young practitioners, as it is extremely valuable on account of its conservative precepts. It is abundantly illustrated by the author's cases.

In bringing our notice of Mr. Bryant's work to a close, we beg leave to reiterate what was stated at the beginning, that, while it is not such a performance as the exigencies of this particular department of science demand at this advanced day, it yet comprises a large amount of useful and instructive matter, conveyed in a plain and simple style, adapted to the comprehension of the humblest capacity. We hope that the author may find leisure to elaborate it into a great treatise that shall be creditable alike to himself and to his country.

